# Early transmission and case fatality of Ebola virus at the index site of the 2013–16 west African Ebola outbreak: a cross-sectional seroprevalence survey

**DOI:** 10.1016/S1473-3099(18)30791-6

**Published:** 2019-04

**Authors:** Joseph W S Timothy, Yper Hall, Joseph Akoi-Boré, Boubacar Diallo, Thomas R W Tipton, Hilary Bower, Thomas Strecker, Judith R Glynn, Miles W Carroll

**Affiliations:** a London School of Hygiene & Tropical Medicine, London, UK; b Research and Development Institute, National Infection Service, Public Health England, Porton Down, Salisbury, UK; c Public Health England/Tropical Medicine Institute Berlin Reference Laboratory, Guéckédou, Guinea; d World Health Organization, Regional Office for Africa, Brazzaville, Republic of Congo; e Institute of Virology, Philipps University, Marburg, Germany

## Abstract

**Background:**

To date, epidemiological studies at the index site of the 2013–16 west African Ebola outbreak in Meliandou, Guinea, have been restricted in their scope. We aimed to determine the occurrence of previously undocumented Ebola virus disease (EVD) cases and infections, and to reconstruct transmission events.

**Methods:**

This cross-sectional seroprevalence survey of the adult population of Meliandou used a highly specific oral fluid test and detailed interviews of all households in the village and key informants. Each household was interviewed, with all members prompted to describe the events of the outbreak, any illness within the household, and possible contact with suspected cases. Information for deceased individuals was provided by relatives living in the same household. Symptoms were based on Ebola virus Makona variant EVD case definitions (focusing on fever, vomiting, and diarrhoea). For antibody testing, we used an Ebola virus glycoprotein IgG capture enzyme immunoassay developed from a previously validated assay. A maximum exposure level was assigned to every participant using a predetermined scale. We used a generalised linear model (logit function) to estimate odds ratios for the association of sociodemographic variables and exposure level with Ebola virus infection. We adjusted estimates for age and maximum exposure, as appropriate.

**Findings:**

Between June 22, and July 9, 2017, we enrolled 237 participants from 27 households in Meliandou. Two households refused to participate and one was absent. All adults in participating households who were present for the interview provided an oral fluid swab for testing, of which 224 were suitable for analysis. In addition to the 11 EVD deaths described previously, on the basis of clinical description and oral fluid testing, we found two probable EVD deaths and eight previously unrecognised anti-Ebola virus IgG-positive survivors, including one who had mild symptoms and one who was asymptomatic, resulting in a case fatality of 55·6% (95% CI 30·8–78·5) for adults. Health-care work (adjusted odds ratio 6·64, 1·54–28·56; p=0·001) and level of exposure (odds ratio adjusted for linear trend across five levels 2·79, 1·59–4·883; p<0·0001) were independent risk factors for infection.

**Interpretation:**

Ebola virus infection was more widespread in this spillover population than previously recognised (21 *vs* 11 cases). We show the first serological evidence of survivors in this population (eight anti-Ebola virus IgG seropositive) and report a case fatality lower than previously reported (55·6% *vs* 100% in adults). These data show the high community coverage achievable by using a non-invasive test and, by accurately documenting the beginnings of the west African Ebola virus outbreak, reveal important insight into transmission dynamics and risk factors that underpin Ebola virus spillover events.

**Funding:**

US Food and Drug Administration, Wellcome Trust, and German Research Council.

## Introduction

Ebola virus disease (EVD) is a haemorrhagic fever characterised by severe, multisystem disease, and a high case fatality.^1^ Ebola viruses are zoonotic pathogens circulating among sylvatic species with scarce direct contact with humans.^2^, ^3^, ^4^ Only 27 distinct zoonotic spillover events among human populations have been identified since the discovery of the virus in 1976.^5^ Owing to the inherent difficulty of surveillance in remote locations, many outbreak investigations rely on retrospective detection, and can overlook mild EVD clinical presentations.^6^, ^7^ Investigation has also been hampered by the absence of reliable immunological tools to quantify past infection.^8^, ^9^ Because of these challenges, there are still knowledge gaps in the natural history and transmission patterns of Ebola virus during early spillover events.

**Research in context**

**Evidence before the study**

We did a systematic review of Ebola virus seroprevalence surveys. We searched PubMed and Web of Science for articles published between Dec 1, 2013, and Dec 31, 2018, using the keywords “Ebola” AND “Meliandou” OR “Guinea”. We selected articles collecting data or describing events at the index site of the outbreak. No language restrictions were used. Despite extensive diagnostic, molecular, and phylogenetic research into the transmission of Ebola virus during the 2013–16 west African outbreak, no quantitative study or diagnostic methods have been employed at the suspected index location of Meliandou village in Guéckédou prefecture of south-eastern Guinea. Previous studies in this area, conducted during the outbreak, were necessarily limited and did not include serological investigation. Past studies on Ebola virus spillover events in other locations have relied on immunoassays with questionable specificity. During the west African outbreak, a new high specificity capture assay capable of detecting anti-Ebola virus immunoglobulins from oral fluid samples demonstrated the occurrence of mild and asymptomatic infections, and facilitated non-invasive serological studies with high population coverage.

**Added value of the study**

This study provides the first evidence of Ebola virus-infected survivors from the index site of the west African outbreak. A thorough retrospective epidemiological study done concomitantly among the resident adult population also greatly expands our understanding of the initiating events including transmission dynamics, probable transmission chains, lower case fatality rates, and the presence of both mild and asymptomatic cases.

**Implications of all the available evidence**

Our conclusions highlight the importance and potential of deploying appropriate quantitative serological tools. The acceptability and specificity of this approach alongside careful epidemiological investigation provide comprehensive understanding of transmission dynamics in Meliandou. These data thoroughly characterise the initiating events of an Ebola virus outbreak and show that it spread further within this community than previously appreciated. They also show that it is possible to access a large proportion of a deeply affected community by building up trust and using an acceptable and non-invasive approach.

Although severe clinical manifestations dominate during human EVD outbreaks, both minimally symptomatic and asymptomatic infections occur, particularly among contacts of cases.^7^, ^10^, ^11^ Evidence for the existence of sub-symptomatic cases includes Ebola virus PCR analysis of contacts of patients in the 1996 Gabon outbreak, who showed signs of viral replication in their blood in the absence of EVD symptoms.^12^ Two surveys^7^, ^10^ done in west Africa suggested that asymptomatic infections are infrequent (2·6% and 7·5% among contacts). The larger of these studies^7^ reliably identified asymptomatic infections using non-invasive oral fluid sampling and a novel anti-Ebola virus IgG capture assay with high specificity (100%, 95% CI 98·9–100) and sensitivity (95·9%, 89·8–98·9).

The true incidence of subclinical infections and their contribution to transmission dynamics are not fully understood.^13^, ^14^ Surveys^8^, ^15^, ^16^ have reported seroprevalence of 0–46% for Ebola virus infection in endemic areas and up to 24% in regions with no previously documented exposure to the virus. The relative contribution of true asymptomatic infections,^17^ exposure to unrecognised filoviruses, or immunoassay cross-reactivity to these findings is not clear.^13^ Comprehensive documentation of Ebola virus spillover incidents will inform understanding on the role of different disease states and the nature of viral spread among exposed communities in the early stages of an outbreak.

The Ebola virus Makona variant (Zaire species), which emerged from Guinea in 2013, caused the largest recorded outbreak of any Ebola virus species in humans (28 625 cases and 11 325 deaths notified)^18^ and stemmed from a single spillover event in the village of Meliandou, Guéckédou prefecture—for which 11 cases were reported, all of whom died.^19^, ^20^, ^21^ This study aims to provide a quantitative description of these initiating events in the absence of any previous serological or diagnostic analyses at this location.^19^, ^22^

## Methods

### Study design and participants

We sought to enrol all family members residing in Meliandou who were aged 18 years or older at the time of the study. Meliandou is a rural village of 30 households living in 75 buildings, all belonging to the Kissi ethno-linguistic group. The village is encircled by a 100–200 m perimeter of forest and is about 12 km by uneven road from the nearest major urban centre (Guéckédou). All residents were checked by village leaders and local health-care workers before registration to confirm that they had been resident during the period of the outbreak in this area (December, 2013, to March, 2014). Information on EVD exposure and symptoms was collected retrospectively. Interviews were done for all households on the basis of qualitative and quantitative approaches used in Sierra Leone, as described previously.^7^, ^23^ Each household was interviewed as a group led by Kissi-speaking field staff using a semi-structured approach. Questions were asked to the entire household, with all members prompted to describe the events of the outbreak, any illness within the household, and possible contact with suspected cases. Two field staff recorded the answers of each participant and, after the interview, all staff discussed the participant responses, recording any reported symptoms and the maximum exposure to a suspected case. A maximum exposure level was assigned to every participant using a predetermined scale (level 1–5; appendix). Information for deceased individuals was provided by relatives living in the same household. Symptoms were based on Ebola virus Makona variant EVD case definitions, with an emphasis on symptoms commonly reported from the earliest clinical reports from the Guinea outbreak (fever, vomiting, and diarrhoea; appendix).^1^, ^19^, ^24^, ^25^ At the end of the study, two further meetings were held with key informants (local health-care worker, community health worker, village chief, and youth leader) to verify reported symptoms and to try to resolve any discordant information between respondents.

Permission for the study was granted by the Guinea Comité National D'Ethique Pour La Recherche en Santé, the ethics committee of the London School of Hygiene & Tropical Medicine, and the UK National Health Service National Research Ethics Service. All participants gave written informed consent before interviewing and sample collection.

### Designation of suspected cases and survivors

Participants were initially denoted as being possible cases on the basis of symptoms reported during interviews (appendix). Possible cases for which clinical symptoms were confirmed by the key informants were reclassified as suspected cases (denoted by S prefix) and possible cases with symptoms that were not corroborated by key informants were designated as unconfirmed (denoted by U prefix). All case designations were determined before anti-Ebola virus IgG results were evaluated. Deaths during the outbreak period following EVD-like symptoms were classified as EVD cases.

### Serological analysis

After each group interview, all adult participants provided an oral fluid swab using Oracol Plus collection devices (Malvern Medical Developments, Worcester, UK). Swabbing was demonstrated by field staff and participants were directly observed firmly rubbing the sponge tip along the upper and lower gums for 90 s. Swabs were sealed and placed on ice in a cool box. The same day, swabs were centrifuged at 1500 g for 10 min to extract oral fluid and stored immediately at −20°C. Swabs were transported to Conakry, Guinea, at a maximum temperature of −15°C and shipped to the UK on dry ice for analysis at Porton Down, UK. Positive controls of oral fluid samples were provided by two PCR-confirmed survivors of Ebola virus infection (based in Guéckédou town) with persistent neutralising anti-Ebola virus IgG titres recorded as part of survivor studies. Serum samples were also acquired from the positive controls, one local negative control, and two suspected EVD survivors from Meliandou. Four UK-based volunteers also provided negative control samples.

We detected human anti-Ebola virus (Zaire) IgG using an enzyme-linked immunosorbent anti-Ebola virus glycoprotein IgG capture assay (Kalon Biological, Guildford, UK) that was developed as a commercially available assay from the validated assay described previously.^7^, ^26^ Samples were thawed for 1–2 days at 4°C and centrifuged at 1500 g for 10 min. The supernatant was removed and diluted 1:2 in sterile transport media: phosphate buffer saline pH 7·4 (Severn Biotech, Kidderminster, UK), 10% fetal calf serum (Gibco, Loughborough, UK), 0·5% gentamicin (Gibco), 0·2% amphotericin B (Sigma-Aldrich, Dorset, UK), and 0·2% Tween 20 (Sigma). All samples were run in duplicate. Optical density was read at 450 nm using a Spectra Max 3 plate reader and is presented as a ratio to the optical density of negative controls on each plate as a normalised optical density.

### Statistical analysis

Epidemiological data were double-entered into EpiData (Odense, Denmark; version 4.2.0.101) and exported into RStudio (version 1.0.13) for analysis. We used a generalised linear model (logit link function) to estimate odds ratios for the association of sociodemographic variables and exposure level with Ebola virus infection (defined as anti-Ebola virus IgG seropositive or died of suspected EVD during the outbreak period). We calculated p values via likelihood ratio tests (packages glm version 3.4.0, epiR version 0.9-96, and lmtest, version 0.9-35). We adjusted estimates for age and maximum exposure, and assessed model assumptions for violations. For additional capture assay analysis to assess the association between sample volume or precipitate and normalised optical density we used unadjusted linear regression and Kruskall-Wallis non-parametric methods.

### Role of the funding source

The sponsor of the study had no role in study design, data collection, data analysis, data interpretation, or writing of the report. The corresponding author had full access to all the data in the study and had final responsibility for the decision to submit for publication.

## Results

Between June 22, and July 9, 2017, we enrolled 237 participants from 27 households in Meliandou. One household was absent and two refused. 237 adults (≥18 years) were interviewed and gave swabs, of whom 117 (49·4%) were men and 120 were women (50·6%), with a mean age of 29·8 years. 38 adults from participating families were absent for survey activities. The most common occupations were subsistence farmers (40·4%), housewives (19·8%), or students or unemployed (23·5%).

32 possible cases were identified via the interviews on the basis of reported symptoms. Of these, 13 people had died and ten were confirmed by key informants to have had EVD-like symptoms at the time, resulting in 23 suspected cases (S1–23). The remaining possible cases with reported symptoms were not confirmed by the key informants (pre-fixed U01–09). Of the 23 suspected cases, three deaths were in children and one survivor with confirmed symptoms was younger than 18 years so no oral fluid sample was collected.

After we checked sample integrity and the volume extracted after centrifugation, 224 of 237 oral fluid samples were available for testing. During assay optimisation, the magnitude of normalised optical density responses in the positive control oral fluid samples (controls with persistent Ebola virus neutralising serum antibody titres) fell below a priori defined cutoffs for seroconversion (appendix).^7^ An alternative seropositive cutoff applied in previous Ebola virus seroprevalence studies^27^, ^28^ was defined as the ratio of the optical density of the test sample to the optical density of four plate-specific negative controls plus three SDs of the mean of the negative controls. To ensure conservative classification of cases, only normalised optical density values above 1·1 were classified as seropositive. Full details of cutoff definitions are in the appendix, with sensitivity analyses varying the cutoff between 2 and 5 SDs above the mean of the negative controls.

**Figure 1:**
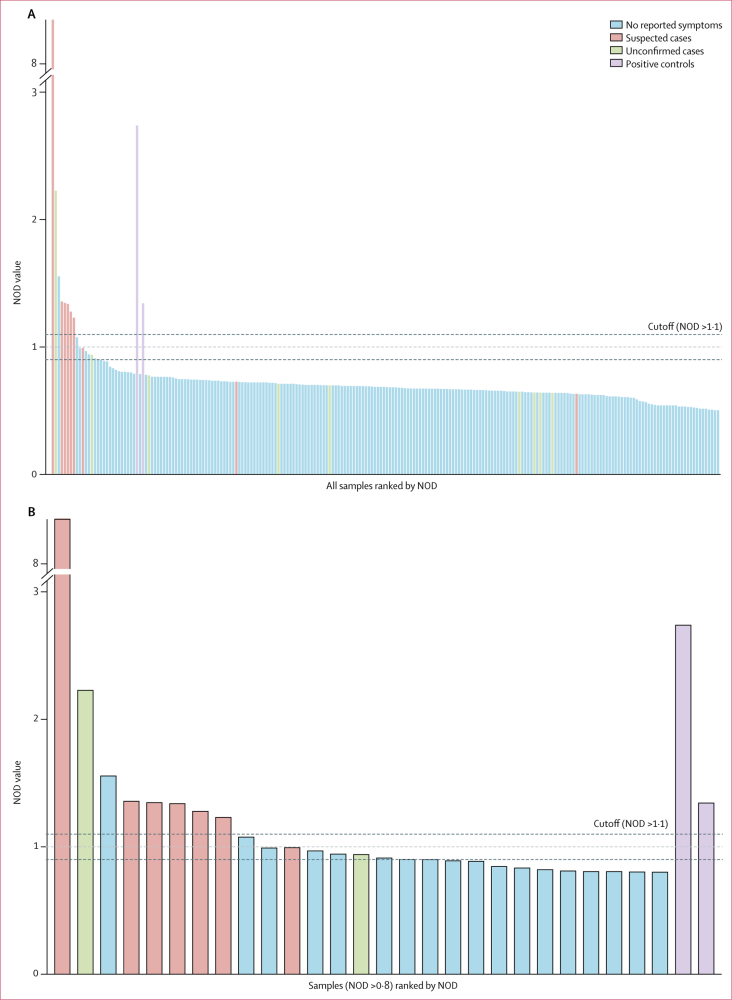
Mean NOD values from oral fluid samples of all study participants using anti-Ebola virus IgG capture assay Samples are ranked by mean NOD value (except the positive controls). All samples were run in duplicate. (A) Mean NOD values of all samples. (B) Mean NOD values of samples with NOD >0·8, for improved resolution. The majority of a priori suspected cases clustered around the highest ranked NOD values. The dashed lines show NOD values of 1·1 and 0·9. NOD values >1·1 were classified as seropositive. NOD=normalised optimal density.

Eight of 224 oral fluid samples were seropositive (3·57%, 95% CI 1·55–6·92) including six of the nine suspected cases in adults (figure 1, table 1); median normalised optical density 1·35), implying 7·29% (4·38–11·27; 18 of 247) of the total adult study population was infected with Ebola virus. Among the eight seropositive adults was one unconfirmed case with self-reported mild nausea, vomiting, and diarrhoea (U01) and high exposure (level 1, direct contact with the corpse of someone with EVD) and one asymptomatic patient (A01) with low exposure (level 4, attended funerals without direct involvement). In the household of the only asymptomatic patient where no other cases were suspected or serologically confirmed, six other family members had their oral fluid sampled and all were negative. Mild or asymptomatic forms of Ebola virus infection represented 11·1% (1·46–36·44; two of 18) of total adult infections.

**Table 1:** Summary oral fluid immunoassay results from all clinically suspected survivors and seropositive oral fluid samples, by case number

	**Case definition**	Exposure level*	**IgG capture assay**†	**Coefficient of variation (all wells)**
**Seropositive cases**				
S11	Suspected	1	2/2	0·04
S12	Suspected	2	2/2	0·01
S13	Suspected	2	2/2	0
S18	Suspected	1	4/4	0·02
S19	Suspected	1	2/2	0·01
S23	Suspected	2	4/4	0·07
A01	Asymptomatic	4	2/2	0·03
U01	Unconfirmed	1	4/4	0·02
**Seronegative clinically suspected cases**				
S14	Suspected	2	0/2	<0·01
S16	Suspected	2	0/2	0·02
S20	Suspected	2	0/2	0·02

All samples with two or more capture assay wells NOD >1·1 were classified as seroconverted and Ebola virus survivors. The number of wells tested and the subsequent number with NOD values greater than 1·1 are shown alongside the coefficient of variation across all capture assay wells tested for each sample. NOD=normalised optimal density. EVD=Ebola virus disease.

* Level 1, contact with EVD corpse; level 2. direct contact with EVD cases or their bodily fluids; level 3, shared household with or cared for EVD case without direct contact; level 4, interaction with EVD cases without contact; and level 5, no known contact. (appendix).

† Data are positive wells/wells tested.

Among unconfirmed cases (U01–09), the median normalised optical density of oral fluid was 0·68 (95% CI 0·67–0·70), compared with 0·71 (0·65–0·94) among all other oral fluid samples. Three suspected cases in which symptoms were confirmed (S14, S16, and S20) remained under the seropositive cutoff with normalised optical density values of 1·0, 0·73, and 0·64, respectively.

**Figure 2:**
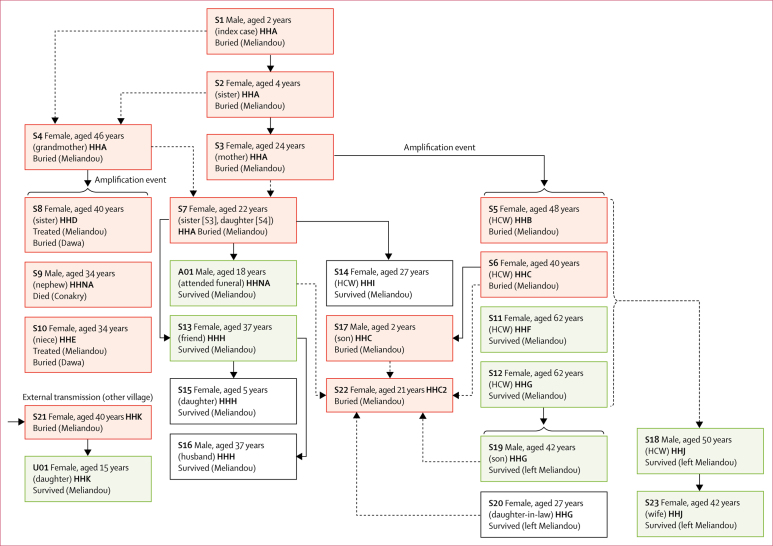
Probable Ebola virus transmission chain based on clinical symptoms of infection and anti-Ebola virus IgG serological results The transmission chain includes all clinically suspected cases for which symptoms were confirmed by key informants (even if seronegative or if serology was not done), all seropositive survivors, and all suspected EVD deaths. The most likely routes of infection are shown, based on the highest level of exposure to a patient with EVD reported during group interviews. Dashed arrows show multiple plausible routes of infection. Red boxes show EVD death. Green boxes show seropositive EVD survivors. White boxes show clinically suspected cases of EVD in patients who were IgG seronegative (S14, S16, and S20) or were too young to collect oral fluid samples (S15). Solid parentheses show multiple potential cases arising from a single source (amplification event). Dashed parentheses towards S18 show uncertainty of the main route of transmission due to extensive level 1–2 exposures to patients S1–S17. HH indicates the individual's household of residence at the onset of symptoms. Relationships described in parentheses within each box describe the relationship of that individual to the probable source of their infection (upstream origin of arrow). HHC and HHC2 denote different dwellings in the same household. Households denoted as HHNA were not given household suffixes because HH was assigned on the basis of the first appearance of symptoms and neither case developed symptoms in Meliandou. EVD=Ebola virus disease. HCW=health-care worker.

By use of interviews and a population of cases defined by immunoassay results, including eight anti-Ebola virus IgG positive survivors, all seronegative patients or suspected cases that were not tested, and 13 suspected EVD deaths (including the three children who died during the outbreak), we generated a transmission chain that affected ten households (figure 2).

The index case arose in December, 2013, in a boy aged 2 years (S1) whose first contact with Ebola virus reservoirs was possibly insectivorous bats.^29^ The infection spread to his sister (S2; aged 4 years) and pregnant mother (S3) who shared a room with S1 while he was symptomatic. All three died within 2 weeks. The mother suffered a spontaneous abortion on the night of her death, during which she was cared for by family members (S4 and S7), local female health-care volunteers (S5, S6, S11, and S12), and a male local health-care worker (S18). All were heavily exposed to blood lost by S3 and all but S18 had close contact with the body immediately after death. All individuals subsequently developed EVD and four died (S4–S7). S4 and S5 caused onward transmission outside Meliandou after attending district hospitals.^19^, ^22^ S4 was the first to develop symptoms but was also exposed to S1–S3 during their illness and after death. In Meliandou, traditional funeral practises involve direct contact with the deceased's body and possessions, including wearing their clothes, and are performed by friends and family of the same sex.

While in hospital, S4 was cared for by several family members (S7–S10) and her body was returned to Meliandou for burial. The next patients, S5 and S6, were the first cases in individuals residing outside the home of the index patient. Both were buried in Meliandou within 2 days of each other. S6's son (S17, aged 2 years) later died with EVD-like symptoms. The funerals of S4–S6 are believed to have spread the infection to other local villages.^19^

The next cases arose in the family of those caring for S4 during her stay in hospital and burial (S7–S10). S7, who also had high exposure to S3 and lived in the index household, was cared for in the home of a friend (S13) while symptomatic. S7 died and was buried in Meliandou. The asymptomatic seropositive patient (A01) attended the funeral of S7 but was not involved in the preparations of the body. The other family members who cared for S4 in hospital (S8–S10) all subsequently developed EVD symptoms. S8 and S10 returned to their home village (Dawa), where they were buried. S9 travelled to Conakry, where he developed symptoms and died.

Although S11 and S12, who were health-care volunteers, were not previously reported as EVD cases, both developed EVD-like symptoms (including fever, vomiting, and diarrhoea). S11's son (S19) cared for her and developed unreported EVD-like symptoms; he subsequently left Meliandou to seek traditional remedies and survived. During the same week, S13 developed symptoms after caring for S7, including contact with body fluids, but survived without admission to hospital.

The local health-care worker (S18) developed EVD-like symptoms, including fever, vomiting, diarrhoea, red eyes, and blurry vision, but survived. He was involved in the care of all affected patients and in several burials. S18 was cared for exclusively by his wife (S23), who was incapacitated with EVD-like symptoms but survived.

The last two suspected EVD deaths in Meliandou (S21 and S22) had safe burials by health authorities and external non-governmental organisations (such burials started after March 10, 2014). S21 was probably exposed to Ebola virus outside Meliandou, while preparing for burial the body of a patient with suspected EVD in another village. Patient U01, who reported mild nausea, vomiting, and diarrhoea, cared for S21 and participated in her funeral. S22, who was the last individual to die, had several possible routes of infection: providing care in the same compound as S6 and S17, interacting with her symptomatic brother (S19), and assisting in the funeral of S7.

None of the first ten patients (S1–10) survived, after which only three patients died and eight survived (in addition to three who were seronegative and a child who was not tested; table 2). Among adults, and including only those who were seropositive as survivors, case fatality was 55·6% (95% CI 30·8–78·5; ten of 18 participants); with the inclusion of three children, this value was 61·9%, excluding potential survivors and mildly symptomatic cases in children.

**Table 2:** Clinically suspected or seropositive cases identified during epidemiological investigation, by case number

	Age, years*	**Sex**	**Household**	**Probable exposure**	**Died**	**Previously reported case**†	**Seropositive**
S1	2	Male	A	Insectivorous bats^29^	Yes	Yes	NA
S2	4	Female	A	Shared bed with S1	Yes	Yes	NA
S3	24	Female	A	Shared room and cared for S1 and S2	Yes	Yes	NA
S4	46	Female	A	Shared bed with S1 and S2; cared for S1, S2, and S3; attended burials of S1–S3	Yes	Yes	NA
S5	48	Female	B	Cared for S3 during spontaneous abortion; participated in burials of S3 and S4	Yes	Yes	NA
S6	40	Female	C	Cared for S3 during spontaneous abortion; participated in burials of S3 and S4	Yes	Yes	NA
S7	22	Female	A2	Cared for S3 during spontaneous abortion; cared for S4 in hospital; participated in burial of S4	Yes	Yes	NA
S8	40	Female	D	Cared for S4 in hospital (sister); participated in burial of S4	Yes	Yes	NA
S9	34	Male	NA	Cared for S4 in hospital (nephew); participated in burials of S3 and S4	Yes	Yes	NA
S10	34	Female	E	Cared for S4 in hospital (niece); participated in burial of S4	Yes	Yes	NA
S11	62	Female	F	Cared for S3 during spontaneous abortion; participated in burials of S3 and S4	No	No	Yes
S12	62	Female	G	Cared for S3 during spontaneous abortion; cared for S4 when ill	No	No	Yes
S13	37	Female	H	Cared for S7 in their home when ill	No	No	Yes
S14	27	Female	I	Cared for S7 with traditional medicine	No	No	No
S15	5	Female	H	Shared home and cared for S13	No	No	NA
S16	37	Male	H	Cared for S13 and S15 when ill	No	No	No
S17	2	Male	C	Shared room with S6	Yes	Yes	NA
S18	50	Male	J	Provided medical care for S1–S17; cared for S3 during spontaneous abortion	No	No	Yes
S19	42	Male	G	Cared for S12 when ill	No	No	Yes
S20	27	Female	G	Cared for S12 when ill	No	No	No
S21	40	Female	K	Prepared body of suspected case in nearby village	Yes	No	NA
S22	21	Female	C2	Transmission not clear; cared for a participated in burial of S6, participated in burial of S7, and shared household with S17	Yes	No	NA
S23	42	Female	J	Cared for S18 when ill	No	No	Yes
U01	15	Female	K	Cared for S21 when ill	No	No	Yes
A01	18	Male	NA	Attended funeral of S7	No	No	Yes

Suspected cases were those in which patients reported at least three symptoms of Ebola virus disease during the outbreak period whose symptoms were confirmed by key informants or who died following Ebola virus disease-like symptoms. Also included are two individuals who were seropositive for anti-Ebola virus IgG whose self-reported mild symptoms were unconfirmed by key informants (U01) or were self-reported asymptomatic (A01). NA=not applicable.

* At the time of the outbreak.

† Reported in publicly available reports from a previous outbreak investigation.^19^, ^22^

Among adult participants (237 interviewed and ten EVD deaths), 92 reported high-level exposure to suspected cases (61 at level 1, 31 at level 2), with a further 45 sharing residence or providing care for a symptomatic patient without direct contact (level 3; table 3). All EVD deaths and seropositive patients with symptomatic EVD reported level 1 or level 2 exposure. The asymptomatic patient (A01) reported level 4 exposure. Strong evidence was found for a linear association of exposure level with Ebola virus infection (cases or deaths, table 3; univariable OR 2·82, 1·66–4·79; p<0·0001). This value was similar after adjustment for age (adjusted OR 2·68, 1·50–4·80) and for age and health-care role (adjusted OR 2·79, 1·59–4·88). The risk of Ebola virus infection was similar after either level 1 or level 2 exposure (table 3).

**Table 3:** Risk factors for Ebola virus infection in the Meliandou population (aged ≥18 years)

		**Cases, n/N**	**Risk (95% CI)**	**Univariate OR**	**p value**	**OR adjusted for age and exposure level**	**p value**
Total		18/247	7·29 (4·38–11·28)	..	..	..	..
Sex							
	Male	4/118	3·39 (0·93–8·45)	1	0·02	1	0·09
	Female	14/129	10·85 (6·06–17·54)	3·47 (1·10–10·86)	..	2·64 (0·80–8·66)	..
Age, years							
	15–25	5/128	3·91 (1·28–2·88)	1·03 (1·00–1·06)	0·05	1·00 (0·97–1·03)	0·82
	25–40	6/62	9·68 (3·63–19·88)	..	..	..	..
	>40	7/57	12·23 (5·08–23·68)	..	..	..	..
Head of family unit							
	Yes	2/44	4·54 (0·56–15·47)	0·56 (0·12–2·51)	0·42	0·31 (0·06–1·56)	0·12
	No	16/203	7·88 (4·58–12·48)	1	..	..	..
Occupation							
	Other or unemployed	8/187	4·28 (1·86–8·23)	1	0·13	1	0·38
	Housewife	5/49	10·20 (3·40–22·23)	2·54 (0·79–8·15)	..	1·76 (0·51–6·06)	..
Health-care role in village (including traditional)							
	No	13/234	5·56 (2·99–9·31)	1	0·0003	1	0·001
	Yes	5/11	45·45 (16·75–76·62)	14·29 (3·85–53·08)	..	6·64 (1·54–28·56)	..
Maximum exposure to EVD case							
	Level 1	11/61	18·03 (9·36–29·98)	2·82 (1·66–4·79)	<0·0001	2·79 (1·59–4·883)	<0·0001
	Level 2	6/31	19·35 (7·45–37·47)	..	..	..	..
	Level 3	0/45	0 (0–7·87)	..	..	..	..
	Level 4	1/87	1·49 (0·03–6·24)	..	..	..	..
	Level 5	0/23	0 (0–14·81)	..	..	..	..

Cases are defined as seropositive for anti-Ebola virus IgG or suspected EVD deaths. We used a generalised linear model with a logit function to calculate crude estimates; p values were calculated via likelihood ratio test. The association with exposure level persisted after additionally adjusting for health-care work (adjusted OR 2·80, 95% CI 1·48–5·31). For sex, the association was lost when health-care workers were removed from the analysis (2·25, 0·58–8·77). Data were missing for occupation (n=11) and health-care role (n=2). EVD=Ebola virus disease. OR=odds ratio.

In the univariable analysis, Ebola virus infection was most common among women, with increasing age, and in those with a formal or informal health-care responsibility in the community (table 3). After adjustment for age and exposure level, health-care responsibilities remained an independent risk factor for Ebola virus infection (OR 6·64, 1·54–28·56; p=0·001). There was no effect of age after adjusting for exposure level, or of sex after health-care workers were removed from the full model (table 3). A sensitivity risk factor analysis with alternative cutoff values is shown in the appendix.

## Discussion

This cross-sectional seroprevalence survey used detailed investigation and non-invasive immunological tools to document Ebola virus transmission at the index site of the largest recorded outbreak of human EVD. Although an outbreak investigation was done in 2014, no diagnostic or immunological methods have been previously used in Meliandou.^19^ Our study adds to past investigations, highlighting a much greater spread of infection, including an increased number of reported deaths (13 *vs* 11) and the identification of eight seropositive survivors. Although initial zoonotic and human-to-human transmission within the index household have already been documented,^19^, ^29^ our account of subsequent events differs notably, particularly in the wider impact of the virus among households not directly related to the index case and the identification of mildly symptomatic and asymptomatic survivors. From a public health perspective, these findings highlight the need for community-sensitive approaches to enhance case finding during spillover events, and the potential of non-invasive tests to aid community participation and gain a better understanding of infection spread.

Identifying survivors enhances our understanding of the transmission dynamics of Ebola virus during this spillover event. After the initial cases in the index family, the virus propagated across numerous households, stemming from a series of high-level exposures to cases in the absence of any preventive interventions. Both traditional funeral practices and contact with symptomatic patients or their body fluids were important in the dissemination of Ebola virus infection, as was also reported in rural areas during the course of the wider outbreak.^30^ The involvement of formal and informal health-care workers in caring for S3 during her spontaneous abortion and death acted as an amplification event, spreading the infection to other households. The early involvement of local health-care workers in the outbreak draws parallels to many previous Ebola virus spillover events.^31^

We found evidence of asymptomatic and minimally symptomatic cases of Ebola virus infection in Meliandou, adding to the evidence on the prevalence of these disease states during the west Africa outbreak.^7^, ^10^ Although we have reported a case as asymptomatic infection, it is challenging to be sure retrospectively that there were no symptoms. Although onward transmission from asymptomatic infection cannot be ruled out, we found no evidence of further infection within the household of the asymptomatic case. Notably, three suspected cases did not self-report symptoms but were identified as symptomatic by key informants. The use of key informants was, therefore, crucial in preventing misclassification of cases as asymptomatic, and is relevant to future studies because of the persistent stigma around EVD.

The case fatality (55·6% in adults, 61·9% including three children aged <18 years) in Meliandou was lower than previously reported (100%).^19^ The value for children could be overestimated owing to the exclusion of infected children with mild or no symptoms. These values are closer to the case fatality of 70·7% among patients with clinically suspected EVD during the early stages of the outbreak.^25^ Despite some Meliandou patients receiving hospital treatment (S4, S5, and S9), Ebola virus was not recognised as the causative agent until March 23, 2014, and patients did not receive targeted treatment.^18^ The case fatality is therefore perhaps lower than might be anticipated given a case fatality of 88·8% among patients not receiving hospital treatment during the initial stages of the outbreak,^1^, ^25^, ^32^ but can be explained by the inclusion of two patients who would not have been recognised at the time. There was some evidence that the case fatality waned over the course of the outbreak in Meliandou.^33^ This observation is unlikely to be due to differences in exposure because, unlike attack rates, intensity of exposure has previously been shown not to correlate with case fatality.^33^ It could be explained through differences in the incubation period (the more susceptible getting ill quicker and being more likely to die), genetic susceptibility, or chance.

The use of a non-invasive immunoassay based on oral fluid to detect anti-Ebola virus IgG was acceptable to the community and ensured high participation. Although the assay was previously validated in a comparable setting,^7^ we saw changes in the assay's performance that required changes in the study cutoff. Between studies, the assay was moved to a commercial manufacturer and a different swab was used, which could explain the differences (appendix). Oral fluid samples used with this capture assay have a reduced titre relative to plasma, yet still reflect the plasma concentration of IgG with a linear relationship.^34^ As this study is the first to report the use of oral fluid to detect anti-Ebola virus IgG over 3 years since initial infection, it is possible that waning IgG titres might contribute to reduced magnitude responses. Previous reports have suggested that serum anti-Ebola virus IgG titres can decrease over time, although this hypothesis has never been comprehensively addressed,^28^ and persistent IgG titres, including Ebola virus neutralising capacity, have been detected 11–40 years after infection.^27^, ^35^ Despite challenges, our study conclusions are robust to variations in the chosen cutoff. Increasing the cutoff to 4 SDs above the mean produced identical results, yet increasing above 5 SDs excluded cases with high live Ebola virus plasma neutralising antibody titre, so appears too stringent. Lowering the cutoff included one further asymptomatic patient, while removal of all cases falling between 2 SD and 5 SD cutoffs only mildly affected the strength of risk factor associations. Although the use of UK-based negative controls could be considered a limitation of the immunoassays, adherence to previous protocols and cutoff sensitivity analysis reinforces the specificity of our findings. Given the loss of several samples owing to low volume oral fluid, we caution future studies against using oral fluid devices that require centrifugation to extract oral fluid.

This study has important limitations. Owing to the time that has elapsed since the outbreak, recall bias is likely, and is only partly offset by using group interviews and key informants. Serology was restricted to adults (aged ≥18 years), so children who had mild or no symptoms might have been missed, which underestimates these disease states.

Our findings from Meliandou provide important documentation of the initiating events of the 2013–16 outbreak of Ebola virus in west Africa. Future research into Ebola virus and other emerging diseases will benefit from the use of acceptable non-invasive sampling to further our knowledge of mild and asymptomatic infection and transmission among populations at risk of Ebola virus spillover events. Such information can improve our understanding of the natural history of Ebola virus and contribute to establishing appropriate and sustainable surveillance systems to prevent communities like Meliandou from suffering the long-term effects of Ebola virus and related outbreaks.
