# Correction to *Lancet Infect Dis* 2019; 19: 429–38

**DOI:** 10.1016/S1473-3099(19)30527-4

**Published:** 2019-11

*Timothy JWS, Hall Y, Akoi-Boré J, et al. Early transmission and case fatality of Ebola virus at the index site of the 2013–16 west African Ebola outbreak: a cross-sectional seroprevalence survey.* Lancet Infect Dis *2019; **19**: 429–38*—In figure 2 of this Article, the dashed line to S22 should have originated from S7, not A01. This change does not affect the written interpretation. This correction has been made to the online version as of Oct 1, 2019.

