# Supplementary material for: Early transmission and case fatality of Ebola virus at the index site of the 2013–16 west African Ebola outbreak: a cross-sectional seroprevalence survey
Source: Lancet Infect Dis. 2019 Apr;19(4):429–38. doi: 10.1016/S1473-3099(18)30791-6 (PMC6437313; doi:10.1016/S1473-3099(18)30791-6)
Supplement: Supplementary appendix [file mmc1.pdf]

THE LANCET Infectious Diseases

Supplementary webappendix

This webappendix formed part of the original submission and has been peer reviewed. We post it as supplied by the authors.

Supplement to: Timothy JWS, Hall Y, Akoi-Boré J, et al. Early transmission and case fatality of Ebola virus at the index site of the 2013–16 west African Ebola outbreak: a cross-sectional seroprevalence survey. *Lancet Infect Dis* 2019; published online Feb 21. http://dx.doi.org/10.1016/S1473-3099(18)30791-6.

Appendix

Contents

Page 2-3: Study case definitions and classification of exposure.

(2) Criteria used to classify individuals' exposure level to Ebola virus**.**

(3) Case definitions used to classify participants as suspected or unconfirmed cases during epidemiological investigation.

Page 4-10: Additional data outlining the use cut-off levels for serological analysis:

(4) Classification of seropositive using original cut-off values.

(5) Validation of internal controls.

(6) Effect of ORF sample volume extracted or precipitation on OD values.

(8-9) Orthogonal corroboration of seropositive status of participant plasma using live Ebola virus neutralising assay.

(10) Assessment of participants plasma vs ORF on capture enzyme immunoassay.

Page 11-15: Sensitivity analysis of findings using alternative seropositive cut-off values.

(11) Assessment of case numbers using different seropositive cut-off values.

(13) Alternative transmission chain using different seropositive cut-off thresholds.

(15) Effect of different seropositive cut-off on risk factor analysis.

Supplementary Information

Section 1: Study case and exposure definitions

Maximum exposure	Criteria of exposure level
**Level 1**	Direct contact with the dead body of a suspected EVD case.
**Level 2**	Providing care for a person with suspected EVD (wet or dry case) that involves direct contact with person or fluids.<br><br>Includes cleaning linen, clothing and sharing a bed.
**Level 3**	Living and sleeping in the same building as a person with suspected EVD while symptomatic.<br><br>Care of a suspected EVD case without direct contact with the person or their used materials (e.g. clothing, linen).
**Level 4**	Interaction and presence with suspected EVD cases without contact (visited household, attended funeral).
**Level 5**	No known contact with any suspected EVD case.<br><br>Avoided suspected EVD cases when symptomatic.

Table 1: **Quantitative exposure level categories used to define individual-level exposure to suspected EVD cases**. All participants were assigned a maximum exposure level following cessation of group interviews and the final exposure was based on feedback from the entire field team (5 members) with separate members either leading discussions or transcribing the interviews.

**Case definitions**	
**Suspected EVD case (S)**	Self-reporting 3 or more EVD-like symptoms during EVD outbreak period (December 2013 – March 2014).<br><br>Suspected cases required confirmation of symptoms by key informants during data verification with key informants including the health post physician.<br><br>All deaths reported with EVD-like symptoms during the outbreak period were denoted as suspected EVD cases.
**Unconfirmed EVD case (U)**	Self-reporting 3 or more EVD-like symptoms during the outbreak period.<br><br>Unconfirmed cases did not have their symptoms independently verified by key informants during data verification interviews.
**List of EVD symptoms**	Fever – Weakness or Tiredness – Headache – Loss of Appetite – Nausea or Vomiting – Abdominal Pain – Diarrhoea – Blood in Stool – Muscle or Joint Pain – Sore Throat with Pain Swallowing – Hiccups – Red Eyes – Blurry Vision – Bleeding Gums - Miscarriage

Table 2: Clinical case definitions for suspected (S) or unconfirmed (U) EVD cases. All clinically suspected cases were designated *a priori* to assessment of oral fluid anti-EBOV IgG.

Section 2: Anti-EBOV IgG seropositive cut-off

Re-assessment of anti-EBOV IgG capture assay seropositive cut-off

Prior to the initiation of the study we planned to use cut-offs from recent validation studies performed during the EBOV outbreak in Sierra Leone using the same anti-EBOV IgG capture assay.[1] This cut-off was the raw optical density (OD) normalised to mean OD of 4 UK negative controls + 0.1OD). Using this approach our results indicated that two samples from surviving Meliandou residents were seropositive, one of whom was a suspected case (S18) while the other had unconfirmed mild EVD-like symptoms (figure 1). Importantly however, one positive control (PCR confirmed) remained under this cut-off (NOD=0.72) yet serum obtained from this participant demonstrated persistent neutralising anti-EBOV titres.

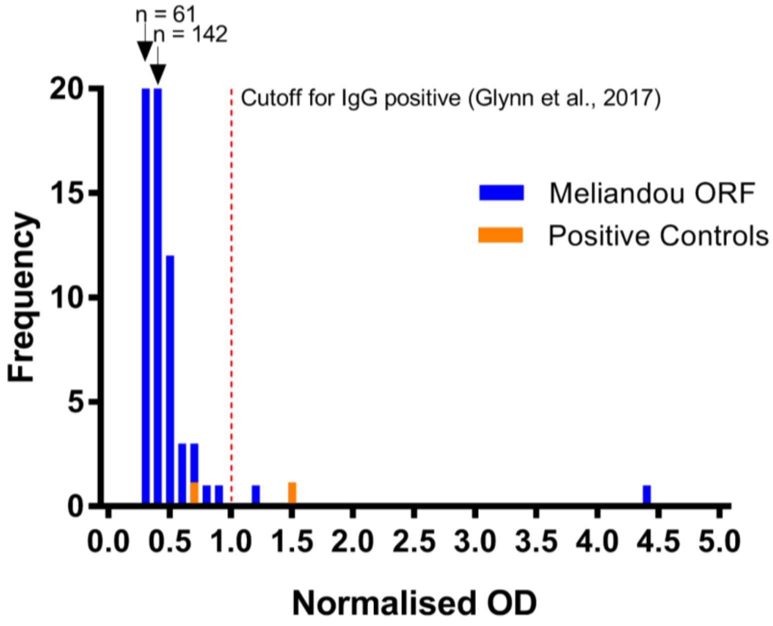

Figure 1: Frequency distribution of normalised optical density of ORF samples presented in relation to assay cut-off described by Glynn et al., (2017). Red line indicates seropositive cut-off used by Glynn et al. (raw 450nm OD normalised to the mean of four plate-specific negative controls plus 0.1) Two participants with an epidemiological basis for EVD surpassed cut-off. This participant was confirmed as possessing EBOV neutralising antibody titre from serum samples acquired three months prior to ORF sampling. These data stimulated re-assessment of the capture assays cut-off.

Since the study by Glynn and colleagues the manufacture of the assay has since been moved to Kalon Biological and changes to the manufacture process were hypothesised to potentially explain the changes in the assay performance. To confirm accurate reproduction of the assay, the manufacturer provided expected values of the internal assay matched by batch number. Comparison of assay controls indicated that the assay was performing to the manufacturer's expectations (table 2).

Comparison of internal control OD values			
	Positive Sera	Negative Sera	Cutoff Calibration
Current study	Mean OD		
> Plate 1	1.31	0.026	0.123
> Plate 2	1.40	0.036	0.131
> Plate 3	1.49	0.023	0.125
> Plate 4	1.32	0.030	0.127
> Plate 5	1.44	0.025	0.139
> Plate 6	1.43	0.037	0.132
Mean	1.40	0.030	0.130
Kalon	Mean OD		
> Plate 1	1.47	0.020	0.122
> Plate 2	1.48	0.026	0.124
Mean	1.48	0.023	0.123

Table 2: Comparison of internal assay controls with expected results based on current assay batch numbers.
Data presented is 450nm OD with 620nm reference OD subtracted from all plates run during current study. Expected values from Kalon internal standards appear comparable.

ORF samples were collected using Oracol Plus swabs, which differed from the study by Glynn et al., in which standard Oracol swabs were used, which require agitation in 1ml transport media rather than centrifugation. The centrifugation required using Oracol Plus devices can result in variable volume extracted between samples. Possible issues relating to differences in extracted ORF were overall volume of fluid extracted and presence of precipitate or visible red/yellow colouration in the ORF sample (figure 2). No evidence was found to suggest that variation in volume of ORF or sample colouration was associated with changes in the OD responses of ORF samples.

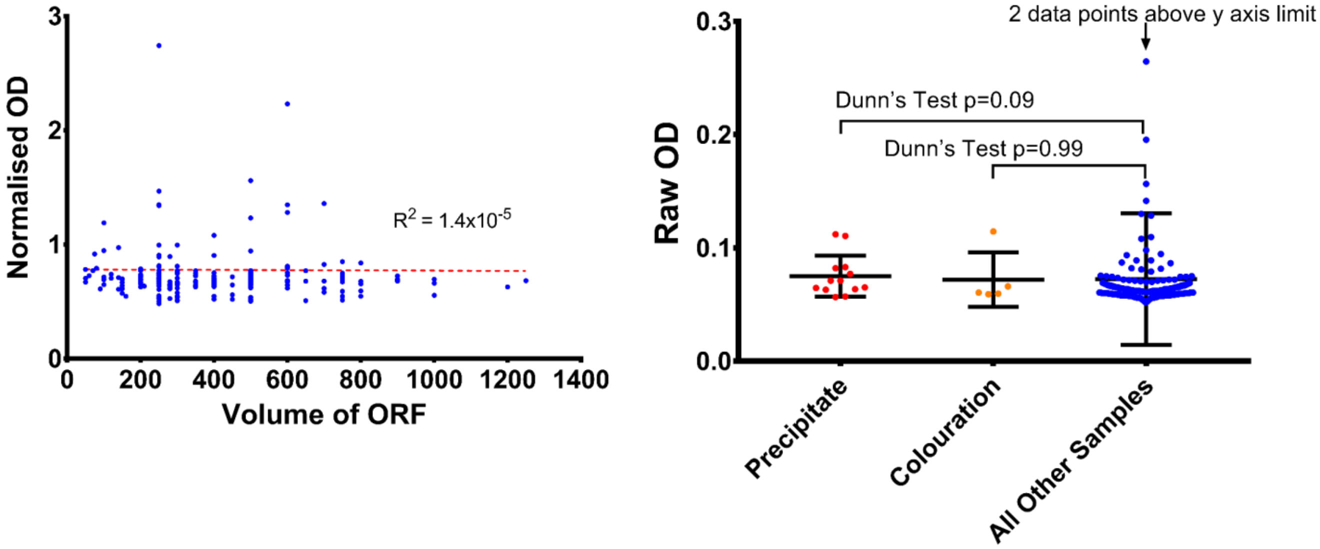

Figure 2: Comparison of ORF volume (µl) and presence of precipitate or colouration on Raw OD values using anti-EBOV IgG capture assay. (A) No evidence for an association of sample volume with raw OD was observed. Simple linear regression p=0.87 and R^2=1.2x10^{-4}. (B) Weak evidence for an association of precipitate in ORF supernatant with raw OD using non-parametric group-wise comparison: Kruskall-Wallis p=0.09, Dunn's test p=0.06 (multiple comparisons). No association was observed between sample colouration and raw OD, Dunn's test p=0.99.

To further validate the use of ORF collected during this study we assessed how the capture assay represented plasma anti-EBOV IgG concentrations from matched study participants. Plasma samples were acquired from two study positive controls, two suspected cases (S18 and S23) and one local negative control. All plasma samples were collected three months prior to ORF sampling and tested using orthogonal methods, whole EBOV ELISA and live EBOV neutralisation assay, as described previously (table 3)[2]. Both Meliandou seropositive cases (S18, S23) and positive controls expressed high anti-Zaire-Guéckédou IgG concentrations and 50% serum neutralising antibody titres >= 1:32. All four plasma samples were then re-tested using the main study capture assay and appeared reactive relative to the negative control plasma sample, yet one positive control and S23 remained close to the cut-off of Glynn et al. (figure 3).

Quantitative comparison of NOD from matched plasma and ORF indicate that ORF samples provide a consistent approximation of plasma IgG levels on the capture assay (table 4). Raw OD values were ~23-28% lower using ORF. Using this assay ORF has previously been shown to accurately reflect plasma anti-EBOV IgG yet with a consistently lower titre versus plasma thus validating the ORF sample used in this study.[3,4]

Study participant	Whole EBOV ELISA tire (Arbitrary ELISA units)	Live EBOV neutralisation assay (50% serum neutralising antibody titre)
Positive Control 1	18201	64
Positive Control 2	14168	76
Meliandou S18	24217	152
Meliandou S23	18315	32
Negative Control	500	4

Table 3: Orthogonal plasma IgG titres from whole Zaire-Guéckédou ELISA and live EBOV neutralising antibody titres from Meliandou study participants

Plasma from two Meliandou survivors denoted as seropositive and both study positive controls underwent orthogonal corroboration using plasma samples. Whole EBOV ELISA data is presented as arbitrary ELISA units and neutralisation assay data as 50% serum live EBO neutralisation assay titre. Both survivors (S18, S23) and positive controls exhibit high anti-EBOV IgG concentration and live EBOV neutralisation titre >=1:32.

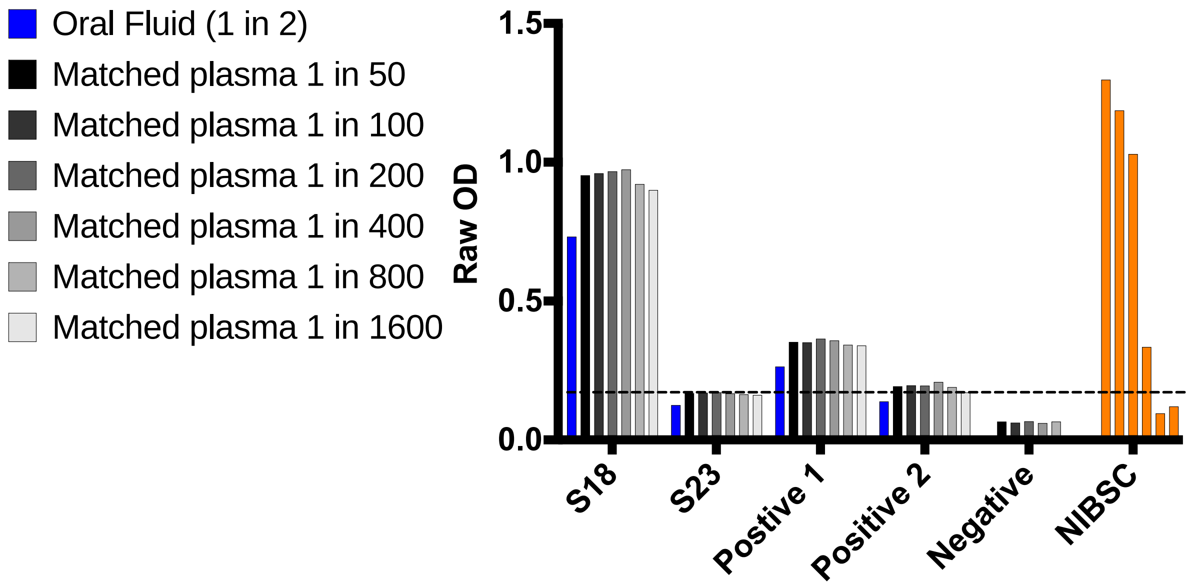

Figure 3: Raw OD values of plasma samples (black-grey bars) collected from Meliandou study participants assessed using anti-EBOV IgG capture assay. Black line indicates plate-specific cut-off used by Glynn et al. and NIBSC standard anti-EBOV reference plasma (highlighted in orange) was also assessed using a 1:10 serial dilution (1:50-1:50x10^5). Blue bars represent the mean raw OD value of matched ORF sample taken from the same participant.

	Raw OD Values			
Sample ID	ORF	Plasma 1:50	Difference (Raw OD)	Proportional Difference
S18	0.732	0.954	0.2218	0.232
S23	0.126	0.169	0.0435	0.257
Positive 1	0.265	0.354	0.0890	0.252
Positive 2	0.139	0.194	0.0545	0.282

Table 4: Comparison of capture assay raw OD values for individually matched ORF (1:2 dilution) and plasma (1:50 dilution). ORF appears to provide a consistent approximation of plasma IgG concentration although at ~23-28% lower OD than plasma in both survivors from Meliandou (S18 and S23) and study positive controls (Positive 1 and Positive 2).

Section 3: Sensitivity Analysis

Given the need to re-define a qualitative seropositive cut-off for anti-EBOV IgG immunological responses in the Meliandou population, we present a sensitivity analysis using a range of cut-off values. Table 5 presents the number of cases defined as seropositive after varying the number of standard deviations from the mean of UK negative controls used to calculate normalised optical density. The consequences of varying the cut-off on the transmission chain is also presented in figure 4.

Cut-off value (Mean of UK controls plus Std Dev.)	Number of seropositive cases	Number of seropositive suspected cases	Number of seropositive unconfirmed cases	Number of seropositive asymptomatic cases
2 * SD	9	6	1	2
**3 * SD**	**8**	**6**	**1**	**1**
4 * SD	8	6	1	1
5 * SD	6	4	1	1

Table 5: Quantification of the number of seropositive cases and clinical classification from the Meliandou study population using different seropositive cut-offs. Alternative cut-off are presented in each row. Each cut-off is defined by the number of standard deviations (2-5) from the mean of negative controls to quantify normalised optical density (NOD).

Using a lower threshold of normalised optical density (NOD >1.1 from the mean plus two standard deviations of UK negative controls) results in one additional seropositive case (denoted as A02). This participant did not report any EVD symptoms and their highest exposure was level 4 when visiting S7 while she was symptomatic then attending her funeral. In neither event did this participant report direct contact with the case or their possessions. Increasing the immunoassay cut-off value by one standard deviation (NOD > 1.1 from the mean plus 4 standard deviations) has no effect on the number of seropositive cases relative to

the main study cut-off. An additional standard deviation increase to the cut-off (NOD > 1.1 from the mean plus 5 standard deviations) causes re-classification of two suspected cases as seronegative. While we were not able to acquire plasma from all participants, we report orthogonal confirmation of neutralising live EBOV and whole EBOV ELISA titres (table 3) for two positive controls and two seropositive Meliandou participants (figure 3). One of these cross-validated cases (S23) exhibits the lowest ORF anti-EBOV IgG NOD response among all 8 seropositive survivors yet also exhibit a plasma EBOV neutralising Ab titre of >1:32. Using the 5 standard deviation threshold excludes this case as seropositive and would appear as too stringent. These data also reinforce that all seropositive cases exhibited a magnitude of ORF anti-EBOV IgG responses greater than case S23. As anti-EBOV IgG ORF responses using this capture assay have repeatedly been shown to correlate with plasma IgG levels in past studies[3,4], and in a limited pool of our data (figure 3), we believe the cut-off we have used is appropriate and specific for past EBOV infection.

To understand the robustness of risk factor analysis to variations in cut-off, we also repeated regression analysis following removal of any participants who could be differentially denoted as seronegative or seropositive using any of the cut-offs in table 5. The analysis was, therefore, repeated with 244 individuals, having removed suspected cases S19 and S23, as well as asymptomatic case A02. The results from logistic regression analysis of this reduced dataset is presented in table 6. As was observed in the original analysis, both exposure level (OR 2.81 95% CI 1.55-5.07) and healthcare work (OR 11.08 95% CI 2.29-53.59) persisted as strong independent risk factors for EBOV infection. There was stronger evidence for the association of sex with infection in this analysis, after also removing of healthcare workers from analysis (OR 4.40 95% CI 0.93-20.90) and being head of a family unit (OR 0.16 95% CI 0.01-0.96). It should be noted, however, that the head of family is characterised by low

exposure-outcome groups and is sensitive to small changes in data, a potential source of sparse data bias.

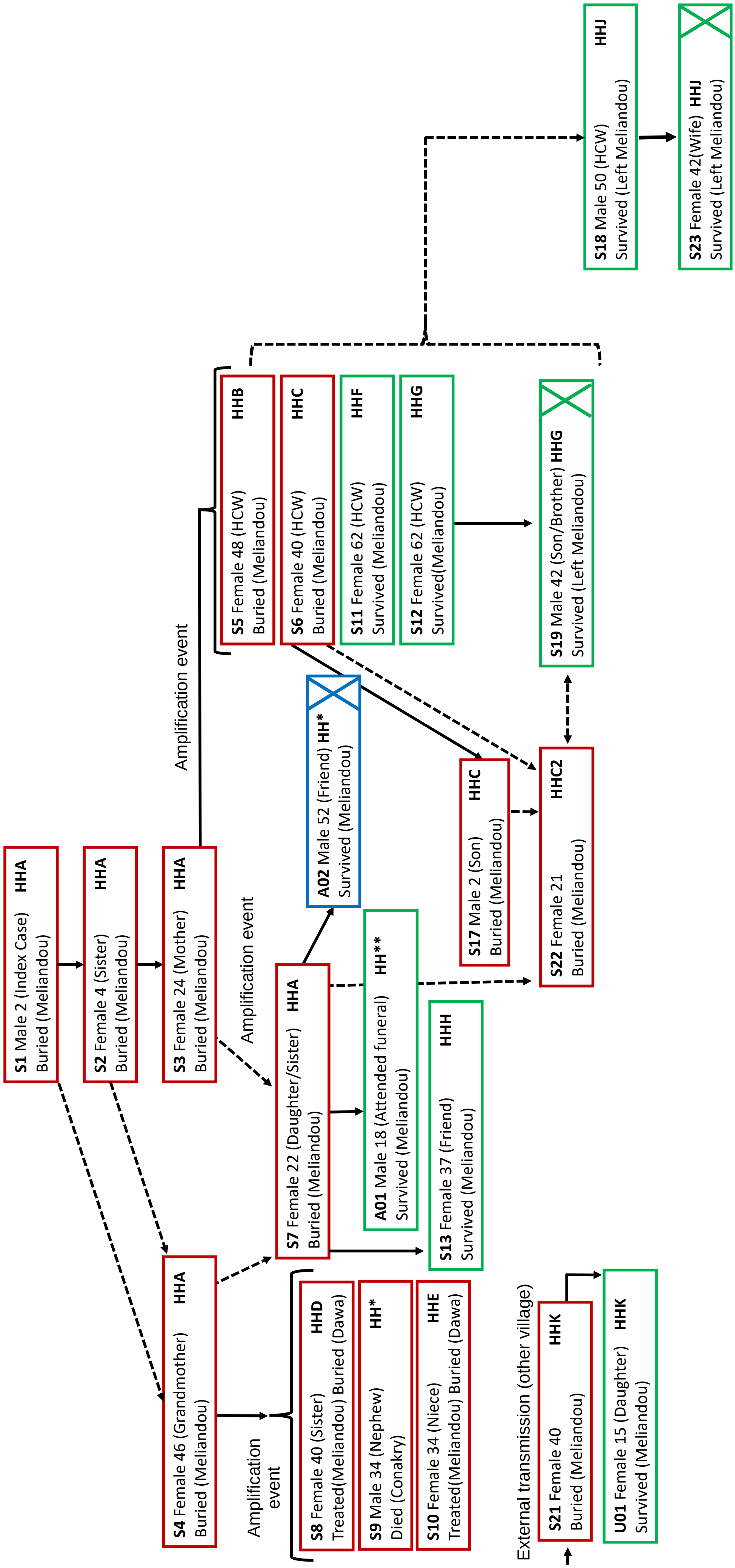
S1 Male 2 (Index Case) Buried (Meliandou) HHA
S2 Female 4 (Sister) Buried (Meliandou) HHA
S3 Female 24 (Mother) Buried (Meliandou) HHA
S4 Female 46 (Grandmother) Buried (Meliandou) HHA
Amplification event
S5 Female 48 (HCW) Buried (Meliandou) HHB
S6 Female 40 (HCW) Buried (Meliandou) HHC
S11 Female 62 (HCW) Survived (Meliandou) HHF
S12 Female 62 (HCW) Survived(Meliandou) HHG
S18 Male 50 (HCW) Survived (Left Meliandou) HHJ
S23 Female 42(Wife) HHJ Survived (Left Meliandou)
S19 Male 42 (Son/Brother) HHG Survived (Left Meliandou)
Amplification event
S7 Female 22 (Daughter/Sister) HHA Buried (Meliandou)
A02 Male 52 (Friend) HH* Survived (Meliandou)
A01 Male 18 (Attended funeral) HH** Survived (Meliandou)
S13 Female 37 (Friend) HHH Survived (Meliandou)
S17 Male 2 (Son) HHC Buried (Meliandou)
S22 Female 21 HHC2 Buried (Meliandou)
Amplification event
S8 Female 40 (Sister) HHD Treated(Meliandou) Buried (Dawa)
S9 Male 34 (Nephew) HH* Died (Conakry)
S10 Female 34 (Niece) HHE Treated(Meliandou) Buried (Dawa)
External transmission (other village)
S21 Female 40 HHK Buried (Meliandou)
U01 Female 15 (Daughter) HHK Survived (Meliandou)

Figure 4: Updated transmission chain based on sensitivity analysis using re-defined anti-EBOV IgG immunoassay cut-off values.

Transmission chain is labelled and colour-coded using the same format as figure 2 of main text. Case A02 is the only additional case included if cut-off is reduced to 2 standard deviations from the mean of controls (blue box). All boxes with a cross in the right-side corner are those excluded if the cut-off is increased to 5 standard deviations from the mean of controls. Note that no difference is observed from the transmission chain in figure 2 of main text if a 4 standard deviation cut-off is applied.

Exposure	Cases	Risk (95% CI)	Univariate OR	p-value	OR adjusted for age and exposure level	p-value
Total	15/244	6.56 (3.79 - 10.43)				
**Sex**						
Male	3/117	10.24 (5.56-16.87)	1	0.01	1	0.06
Female	13/127	2.56 (0.53-7.31)	4.33 (1.20-15.62)		3.21 (0.85-12.05)	
**Age**						
15-25	5/128	3.91 (1.28-2.88)	1.03 (1.00-1.06)	0.09	1.00 (0.97-1.03)	0.98
25-40	6/62	9.68 (3.63-19.88)				
40+	5/54	9.26 (3.08-20.30)				
**Head of family unit**						
Yes	1/43	2.33 (0.06-12.29)	0.30 (0.04 - 2.29)	0.17	0.16 (0.01-0.96)	0.04
No	15/201	7.46 (4.24-12.01)	1			
**Occupation**						
Other profession or unemployed	7/185	3.78 (1.53-7.64)	1	0.09	1	0.28
Housewife	5/49	10.20 (3.40-22.23)	2.89 (0.88-9.54)		2.02 (0.57-7.14)	
**Healthcare role in village (including traditional)**						
No	9/231	3.90 (1.80-7.27)	1	<0.001	1	0.003
Yes	5/11	45.45 (16.75-76.62)	20.56 (5.27-80.18)		11.08 (2.29-53.69)	
**Maximum Exposure**						
Level 1	10/60	16.67 (8.29-28.52)	2.80 (1.60-4.89)	<0.001	2.81 (1.55-5.07)	<0.001
Level 2	5/30	16.67 (5.64-34.72)				
Level 3	0/45	0 (0-7.87)				
Level 4	1/86	1.16 (0.03-6.31)				
Level 5	0/23	0 (0-14.81)				

Table 6: Univariable and multivariable sensitivity risk factor analysis following removal of case S19, S23 and A002 (n=244). Odds ratios are estimated for EBOV infection as the outcome measure. Crude and adjusted estimates were made using a generalised linear model with logit link function and p-values were calculated using likelihood ratio tests. Multivariable estimates were adjusted for age and maximum exposure. The association with exposure level persisted after also adjusting for healthcare work (adjusted OR 2.82 95% CI 1.41-5.56). For sex, healthcare workers were removed from analysis and the association remained (adjusted OR 4.40 95% CI 1.10-29.42) Data was missing for occupation (n=10) and healthcare role (n=2).

References

1 Glynn JR, Bower H, Johnson S, *et al.* Asymptomatic infection and unrecognised Ebola virus disease in Ebola-affected households in Sierra Leone: a cross-sectional study using a new non-invasive assay for antibodies to Ebola virus. *Lancet Infect Dis* 2017; **3099**: 1–9.

2 Agnandji ST, Huttner A, Zinser ME, *et al.* Phase 1 Trials of rVSV Ebola Vaccine in Africa and Europe. *N Engl J Med* 2016; **374**: 1647–60.

3 Lambe T, Rampling T, Samuel D, *et al.* Detection of vaccine induced antibodies to Ebola virus in Oral Fluid. *Open Forum Infect Dis* 2016; **44**: ofw031.

4 Tedder RS, Samuel D, Dicks S, *et al.* Detection, characterization, and enrollment of donors of Ebola convalescent plasma in Sierra Leone. *Transfusion* 2018; **58**: 1289–98.
